# Longitudinal trajectories of comorbid PTSD and depression symptoms among U.S. service members and veterans

**DOI:** 10.1186/s12888-019-2375-1

**Published:** 2019-12-13

**Authors:** Richard F. Armenta, Kristen H. Walter, Toni Rose Geronimo-Hara, Ben Porter, Valerie A. Stander, Cynthia A. LeardMann, for the Millennium Cohort Study Team, Lauren Bauer, Satbir Boparai, Ania Bukowinski, Carlos Carballo, Felicia Carey, James Davies, Alex Esquivel, Gia Gumbs, Isabel Jacobson, Zeina Khodr, Claire Kolaja, William Lee, Gordon Lynch, Denise Lovec-Jenkins, Rayna Matsuno, Deanne Millard, Chiping Nieh, Anet Petrosyan, Jacqueline Pflieger, Chris Phillips, Teresa Powell, Sabrina Richardson, Anna Rivera, Beverly Sheppard, Steven Speigle, Evelyn Sun, Lexi Takata, Daniel Trone, Daniel Vaughan, Jennifer Walstrom, Steven Warner, Kelly Woodall

**Affiliations:** 1 Department of Kinesiology, College of Education, Health, and Human Services, California State University, San Marcos, CA USA; 2 Leidos, 11951 Freedom Drive, Reston, VA 20190 USA; 3 Deployment Health Research Department, Naval Health Research Center, San Diego, 140 Sylvester Road, San Diego, CA 92106-3521 USA; 4 Health and Behavioral Sciences Department, Naval Health Research Center, San Diego, CA 92106-3521 USA

**Keywords:** Comorbidity, Posttraumatic stress disorder, Major depressive disorder, Military personnel, Veterans

## Abstract

**Background:**

Posttraumatic stress disorder (PTSD) often co-occurs with other psychiatric disorders, particularly major depressive disorder (MDD). The current study examined longitudinal trajectories of PTSD and MDD symptoms among service members and veterans with comorbid PTSD/MDD.

**Methods:**

Eligible participants (*n* = 1704) for the Millennium Cohort Study included those who screened positive at baseline for both PTSD (PTSD Checklist–Civilian Version) and MDD (Patient Health Questionnaire). Between 2001 and 2016, participants completed a baseline assessment and up to 4 follow-up assessments approximately every 3 years. Mixture modeling simultaneously determined trajectories of comorbid PTSD and MDD symptoms. Multinomial regression determined factors associated with latent class membership.

**Results:**

Four distinct classes (chronic, relapse, gradual recovery, and rapid recovery) described symptom trajectories of PTSD/MDD. Membership in the chronic class was associated with older age, service branch, deployment with combat, anxiety, physical assault, disabling injury/illness, bodily pain, high levels of somatic symptoms, and less social support.

**Conclusions:**

Comorbid PTSD/MDD symptoms tend to move in tandem, and, although the largest class remitted symptoms, almost 25% of participants reported chronic comorbid symptoms across all time points. Results highlight the need to assess comorbid conditions in the context of PTSD. Future research should further evaluate the chronicity of comorbid symptoms over time.

## Background

Posttraumatic stress disorder (PTSD) is a psychiatric disorder that can result from exposure to a traumatic event in both military and civilian populations [1]. PTSD is especially likely to co-occur with other psychiatric disorders [2], referred to as comorbidity. PTSD is more commonly accompanied by other psychiatric disorders than existing alone [3], including in US active duty service members [4, 5]. Among the psychiatric disorders, major depressive disorder (MDD) is highly comorbid with PTSD (52% [6];) and the disorder most frequently comorbid with PTSD among active duty service members [4, 7]. PTSD and MDD comorbidity is associated with greater negative sequelae for individuals with both disorders in comparison to those with either disorder alone, including reduced perceived social support [8], poorer occupational and social functioning [9], greater health care utilization [10–13], and a significantly elevated risk for persistent PTSD symptoms and suicide [14–17].

Despite the prevalence of comorbid PTSD and MDD, and the deleterious outcomes related to this comorbidity, little is known about how these symptoms fluctuate concurrently over time and what factors are associated with comorbid symptom trajectories. A growing literature has detailed heterogeneous trajectories of PTSD over time following traumatic exposure [18]. Generally, these studies have found four separate trajectories describing resilience, chronicity, recovery, and delayed onset. Resilience is the most common trajectory, particularly among military members. Previous studies have found fewer than 20% of service members categorized in all other trajectories combined [19–21]. Results from these previous studies, which include such a large proportion of resilient individuals, may mask important heterogeneity among individuals with high symptomatology. For example, in these studies, typically only one trajectory includes individuals with high symptomology at baseline. Therefore, limited information is available about potential heterogeneity among clinical populations. Specifically, studies have not directly addressed symptom trajectories among individuals with comorbid PTSD and MDD [22–25]. Thus, given the high prevalence and co-occurrence of MDD among individuals with PTSD, and the greater impairment associated with comorbid PTSD and MDD, it is important to understand prognostic symptom patterns among this subgroup. The current study aimed to fill this knowledge gap by identifying comorbid symptom trajectory classes among a representative sample of service members and veterans with probable comorbid PTSD and MDD. A second objective of this study is to determine factors predictive of symptom trajectories to be able to identify those who may have a more persistent course of symptoms potentially warranting further intervention.

## Methods

### Study population and data sources

Launched in 2001, the Millennium Cohort Study is the largest longitudinal study of military personnel and veterans [26, 27]. Enrolled in phases, service members are recruited from all service branches and components (i.e., active duty, Reserves, National Guard) to examine the long-term health of military service. Following enrollment, participants are requested to complete self-administered surveys (online or paper) approximately every 3 years. The Millennium Cohort survey assesses physical, behavioral, and mental health, as well as military and non-military life experiences. Detailed descriptions of the methods of this study have been published elsewhere [26–28].

The current study included Millennium Cohort participants who enrolled in 2001, 2004, or 2007. Eligible participants must have screened positive for both PTSD and MDD at baseline and completed at least 2 additional follow-up questionnaires, resulting in a final study population of 1704 participants. This study was approved by the institutional review board at the Naval Health Research Center, and all participants provided voluntary, written informed consent.

### Measures

### PTSD and MDD

Based on eligibility criteria, participants must have screened positive for PTSD and MDD at baseline. PTSD was assessed with the PTSD Checklist–Civilian Version (PCL-C) [29]. PTSD screening criteria were consistent with diagnostic criteria of the *Diagnostic and Statistical Manual of Mental Disorders, Fourth Edition, Text Revision (DSM-IV-TR)* [30] of endorsing “Moderately” or higher on at least 1 intrusion item, 2 hyperarousal items, and 3 avoidance items. This DSM-IV-TR criterion for scoring the PCL-C has been shown to correspond to a total cutoff of 44 in military personnel, a sufficiently high threshold for estimating prevalence in a population based study [31, 32]. MDD was measured using the 8-item depression scale from the Patient Health Questionnaire (PHQ-8) [33], consistent with the *DSM-IV-TR* criteria of endorsing at least 5 items as “More than half the days” or higher, in which 1 of the symptoms was anhedonia or depressed mood. Previous studies have found substantial agreement between the PHQ-8 and the PHQ-9 [34].

The outcome of interest in this study was comorbid PTSD and MDD symptom severity, where higher scores indicated increasing morbidity. At each follow-up, PTSD symptom severity was assessed and calculated as the sum of the 17 PTSD Checklist–Civilian Version items, with scores ranging from 17 to 85. MDD symptom severity was also evaluated at each time point and scored as the sum of the PHQ-8 items, which ranged from 0 to 24. Higher scores are suggestive of higher symptom severity for both measures.

### Covariates

Covariates were included based on factors associated with PTSD and MDD identified in prior research [17, 35–38].

#### Demographic and military characteristics

Age, sex, race/ethnicity, service branch, service component, and pay grade were obtained from personnel records maintained by the Defense Manpower Data Center upon study enrollment (baseline visit). Marital status and education were self-reported at baseline.

#### Deployment and combat deployment

Deployment history prior to baseline was assessed using electronic deployment data, obtained from the Defense Manpower Data Center, in combination with self-reported combat experience measured at baseline. Participants were categorized as deployed, deployed without combat, or deployed with combat. Those who deployed were considered to have experienced combat if they reported personal exposure to at least 1 of the following: witnessing death, physical abuse, dead and/or decomposing bodies, maimed soldiers or civilians, or prisoners of war or refugees.

#### Life events

Childhood trauma of physical abuse, sexual abuse, verbal abuse, and neglect before the age of 18 was assessed on the 2016 survey using items from the Juvenile Victimization Questionnaire [39]. Each experience was categorized as happening never, once or more, or prefer not to answer. Sexual assault, physical assault, and disabling injury/illness were each assessed as individual binary covariates using 1 item each. Other life events, including sexual harassment, divorce, and financial stress, were summed and combined into a single categorical variable (range, 0–3) [17]. These stressful life events are broadly based on a modified version of the Social Readjustment Rating Scale-Revised [40] with items that were considered criterion A kept as separate items and those items not considered criterion A collapsed into one variable as demonstrated by others [40].

#### Behavioral characteristics, mental health, and comorbid conditions

Behavioral characteristics and mental health conditions were based on self-reported data at baseline. Body mass index, calculated from self-reported height and weight, was classified as healthy (<25.0 kg/m^2^), overweight (25.0–29.9 kg/m^2^), and obese (> 29.9 kg/m^2^). Smoking status was categorized as never smokers (smoked less than 100 cigarettes), former smokers (smoked at least 100 cigarettes but quit successfully), and current smokers (smoked at least 100 cigarettes but did not report quitting). Alcohol problems were identified as an affirmative response to any of the 5 problematic drinking behaviors based on the related Patient Health Questionnaire (PHQ) alcohol module (e.g., “drank alcohol even though a doctor suggested that you stop drinking because of a problem with your health” or “drove a car after having several drinks or after drinking too much”) [41]. Sleep duration was determined by self-reported hours of sleep on average each night. Social support was based on a single item: “During the last 4 weeks, how much have you been bothered by having no one to turn to when you have a problem,” with 3 response options: “not bothered,” “bothered a little,” and “bothered a lot” [17].

Using the standardized PHQ scoring mechanisms, other anxiety syndrome was assessed at baseline using 6 generalized anxiety items [41]. Bodily pain was assessed using the corresponding profile from the Medical Outcomes Study ShortForm 36-item Survey for Veterans [42]. Scores ranged from 0 to 100 with a higher score indicating more bodily pain and were rescaled on a 0 to 4 scale (where 4 represented highest level of pain) to aid interpretability. Somatic symptoms were identified using the PHQ-15. Items were summed (range, 0–30; α = 0.82) [43] and collapsed into the 3 standard categories (0–9, 10–14, and ≥ 15) [44].

### Statistical analysis

Mixture modeling was used to develop trajectory classes that simultaneously captured non-linear trajectories of PTSD and MDD symptoms. This means that every estimated class had 2 trajectories associated with it; 1 for PTSD and 1 for MDD. Mean PTSD and MDD symptoms were allowed to vary across classes. Mixture modeling estimation used a robust maximum likelihood estimator, which uses full information maximum likelihood to account for missing outcome data and estimate values based on all available data [45]. Unadjusted models with 2 to 7 latent classes were examined to determine the ideal number of classes for the final model. The optimal number of classes was selected using a combination of Bayesian information criterion, Lo-Mendell-Rubin adjusted likelihood ratio test, bootstrap likelihood ratio test, and interpretability of trajectory classes [46, 47]. Additionally, all trajectory classes were required to have at least 2% of the total study population to prevent unstable trajectory classes. Covariates predicting latent classes were entered directly into the mixture model to determine an adjusted model. Missing covariate data were assigned a special missing code to prevent the listwise deletion of incomplete observations. In addition to demonstrating associations with mental health trajectories, similarity between the adjusted and unadjusted models indicate model stability. To ensure that related PTSD and MDD trajectories were not a result of the overlapping items on the two scales, we ran a sensitivity analysis that removed the overlapping items before scoring (i.e., items on sleep, loss of interest, and trouble concentrating). The scales with removed items were rescored and mixture modeling was conducting following the same methods described above. Multicollinearity was assessed using variance inflation factors (VIF) with a VIF > 4 indicating collinearity between covariates; no covariates were above this threshold. All data manipulation was performed using SAS, version 9.4 (SAS Institute Inc., Cary, North Carolina), and all mixture models were performed using Mplus, version 8.0.

## Results

Of the 1704 study participants, the majority were male (60.9%), non-Hispanic white (71.3%), college educated (74.3%), in the Army (57.9%), active duty (66.9%), and enlisted (93.4%) with a mean age of 29.1 years (standard deviation, 8.6). A total of 30.3% of participants deployed before their baseline assessment; of those, 85.5% self-reported combat experience.

Table 1 shows model fit and selection criteria for 2 to 7 latent classes of comorbid PTSD/MDD, as well as unadjusted percentages of participants in each class. A 4-class solution was selected to represent trajectories of probable comorbid PTSD/MDD using a combination of criteria, including a low Bayesian information criterion, high entropy, significant Lo-Mendell-Rubin adjusted likelihood ratio test of the 5-class solution did not significantly improve model fit beyond the 4-class model, and interpretability of classes (Table 1). The average posterior probabilities of membership in classes 1, 2, 3, and 4 were 0.91, 0.97, 0.80, and 0.91, respectively, indicating distinct classes. Class 1, which included 31.1% of participants, was defined as the rapid recovery class. Participants in this class remitted symptoms by the first follow-up and remained at a low level of symptom severity for both conditions throughout the study period. Class 2, which included 26.1% of participants, was considered chronic. The chronic class showed the highest level of symptom severity for both PTSD and MDD throughout the study period. Class 3, which included 24.5% of participants, was categorized as relapse. Participants in the relapse class had a lower level of symptom severity for both PTSD and MDD at their first follow-up, but their symptoms steadily worsened again over the remainder of the study period. Class 4, the smallest class with 18.3% of participants, was classified as the gradual recovery class. For those in the gradual recovery class, both PTSD and MDD symptoms steadily remitted throughout the study period.

**Table 1:** Model Fit and Selection Criteria for 2 to 7 Latent Classes for Comorbid Posttraumatic Stress Disorder and Major Depressive Disorder

	2 classes	3 classes	4 classes^a^	5 classes	6 classes	7 classes
BIC	90,759	89,985	**89,612**	89,391	89,228	89,089
*P* from LMR	< 0.001	< 0.001	**0.010**	0.300	0.276	0.240
*P* from BLRT	< 0.001	< 0.001	**< 0.001**	< 0.001	< 0.001	< 0.001
Entropy	0.854	0.807	**0.783**	0.779	0.767	0.760
Percentage in class 1	52.7	38.6	**31.1**	30.0	26.7	24.2
Percentage in class 2	47.3	35.5	**26.1**	23.0	22.9	16.3
Percentage in class 3		25.9	**24.5**	17.3	13.4	14.5
Percentage in class 4			**18.3**	15.7	13.1	12.1
Percentage in class 5				14.0	12.6	12.1
Percentage in class 6					11.4	10.9
Percentage in class 7						9.8

*BIC* Bayesian information criterion, *BLRT* bootstrap likelihood ratio test, *LMR* Lo-Mendell-Rubin adjusted likelihood ratio test

^a^ Selected class solution is bolded

**Fig. 1:**
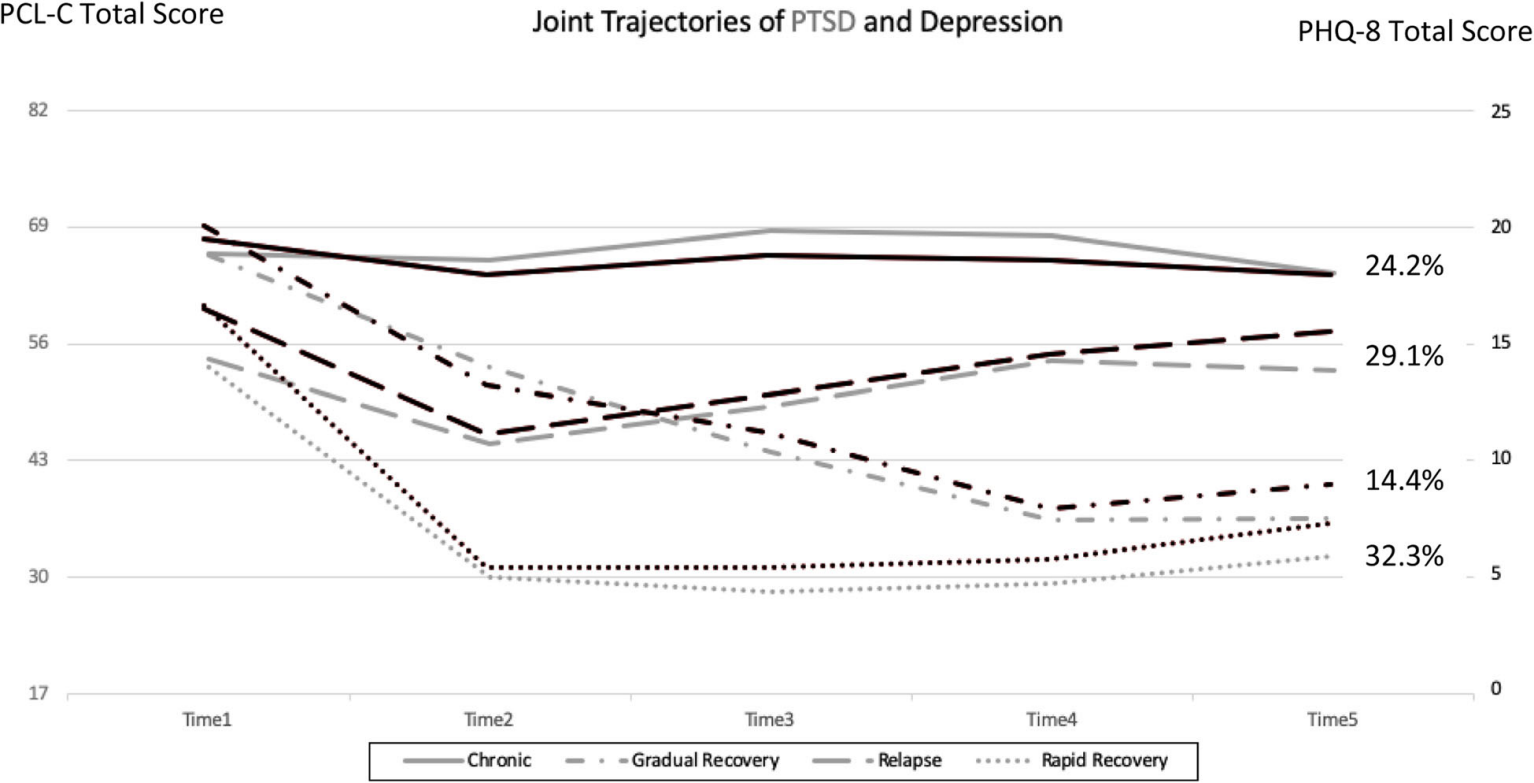
Joint trajectories of comorbid posttraumatic stress disorder (PTSD) and major depressive disorder (MDD) adjusted for covariates in the model. Black indicates trajectories for MDD and gray indicates trajectories for PTSD. All participants screened positive for comorbid PTSD/MDD at time 1. Each time point is approximately 3 years apart. Abbreviations: PCL-C, PTSD Checklist–Civilian Version; PHQ-8: Patient Health Questionnaire 8-item depression scale. Gray line: PTSD. Black line: MDD

Figure 1 shows the joint trajectories of comorbid PTSD/MDD in the fully adjusted model. Class trajectories did not noticeably change after adjusting for covariates in the model indicating stability of the 4-class solution [46]. Further, in the sensitivity analysis that removed overlapping items from the scale, the trajectories were nearly identical to the original trajectories with no items removed (Additional file 1: Figure S1). In adjusted models, participants in the chronic class had higher odds of being older, Hispanic, less educated, and deployed with combat compared with those in the rapid recovery class (Table 2). They also had higher odds of having anxiety, inadequate social support, having been physically assaulted, a disabling injury or illness, and elevated bodily pain or somatic symptoms. Membership in the relapse class was associated with higher odds of combat deployment, obesity, and childhood physical abuse compared with the rapid recovery class in adjusted models. Finally, in adjusted models, those in the gradual recovery class had higher odds of having anxiety, inadequate social support, and a disabling injury or illness; in addition, they had higher odds of sleeping > 9 h per night compared with 7 to 9 h a night compared with those in the rapid recovery class(Table 2).

**Table 2:** Adjusted Odds Ratios of Characteristics Associated With Membership in the Chronic, Relapse, and Gradual Recovery Classes Compared With the Rapid Recovery Class (*n* = 1704)

	*n*^a^	%	Chronic (24.2%)		Relapse (29.1%)		Gradual Recovery (14.4%)	
			aOR	95% CI	aOR	95% CI	aOR	95% CI
Demographics								
Age^b^								
10-year increment	29.1 (8.6)		**1.67**	**1.21, 2.30**	1.01	0.77, 1.31	1.31	0.76, 2.25
Sex								
Male	1038	60.9	Referent		Referent		Referent	
Female	666	39.1	0.83	0.51, 1.33	0.93	0.63, 1.39	1.14	0.57, 2.25
Race/ethnicity								
Non-Hispanic white	1215	71.3	Referent		Referent		Referent	
Non-Hispanic black	210	12.3	1.51	0.85, 2.68	1.32	0.79, 2.20	1.63	0.72, 3.65
Hispanic	161	9.5	**2.21**	**1.16, 4.16**	0.76	0.41, 1.40	2.06	0.60, 7.00
Other	118	6.9	1.38	0.66, 2.89	0.78	0.38, 1.61	0.67	0.08, 5.67
Marital status								
Single or never married	624	36.6	Referent		Referent		Referent	
Currently married	744	43.7	0.90	0.57, 1.42	1.07	0.71, 1.61	0.89	0.48, 1.63
Widowed, divorced, or separated	336	19.7	1.09	0.60, 1.97	1.45	0.83, 2.54	0.81	0.33, 1.99
Education								
Bachelor’s degree or higher	256	15.0	Referent		Referent		Referent	
Some college	1011	59.3	2.01	0.97, 4.14	1.14	0.66, 1.96	1.97	0.32, 11.96
High school or less	436	25.6	**2.62**	**1.19, 5.73**	0.94	0.51, 1.73	2.05	0.30, 13.76
Military characteristics								
Service branch								
Army	987	57.9	Referent		Referent		Referent	
Navy, Coast Guard	281	16.5	**0.49**	**0.26, 0.94**	0.69	0.43, 1.10	1.76	0.70, 4.39
Marine Corps	191	11.2	1.18	0.65, 2.13	0.87	0.48, 1.56	1.04	0.49, 2.18
Air Force	245	14.4	0.59	0.30, 1.15	0.74	0.45, 1.22	1.00	0.46, 2.16
Service Component								
Active duty	1140	66.9	Referent		Referent		Referent	
Reserve, National Guard	564	33.1	0.90	0.59, 1.35	0.70	0.48, 1.02	0.91	0.50, 1.67
Pay grade								
Officer	112	6.6	Referent		Referent		Referent	
Enlisted	1592	93.4	0.90	0.33, 2.42	1.91	0.82, 4.40	0.73	0.18, 2.91
Deployment/Combat^c^								
None	1186	69.6	Referent		Referent		Referent	
Deployed, no combat	75	4.4	1.08	0.41, 2.84	0.72	0.34, 1.49	1.18	0.44, 3.15
Deployed with combat	442	25.9	**4.28**	**2.58, 7.07**	**1.63**	**1.05, 2.52**	1.75	0.89, 3.44
Stressful life events								
Childhood physical abuse								
Never	604	35.5	Referent		Referent		Referent	
Once or more	695	40.8	1.08	0.61, 1.88	**2.42**	**1.49, 3.92**	0.92	0.42, 2.01
Prefer not to answer	61	3.6	3.40	0.72, 15.73	2.11	0.55, 7.96	4.34	0.63, 29.6
Childhood sexual abuse								
Never	971	57.0	Referent		Referent		Referent	
Once or more	321	18.8	1.32	0.75, 2.30	0.76	0.46, 1.24	1.28	0.50, 3.24
Prefer not to answer	69	4.1	0.63	0.23, 1.71	0.42	0.16, 1.07	0.65	0.14, 2.90
Childhood verbal abuse								
Never	622	36.5	Referent		Referent		Referent	
Once or more	676	39.7	1.37	0.79, 2.35	1.38	0.83, 2.30	1.79	0.75, 4.25
Prefer not to answer	64	3.8	1.28	0.34, 4.72	**3.44**	**1.09, 10.73**	0.59	0.07, 4.55
Childhood neglect								
Never	963	56.5	Referent		Referent		Referent	
Once or more	346	20.3	1.57	0.87, 2.84	1.19	0.70, 2.03	0.72	0.27, 1.92
Prefer not to answer	56	3.3	2.50	0.59, 10.34	0.90	0.26, 3.02	1.43	0.12, 16.46
Sexual assault								
Not endorsed	1291	75.8	Referent		Referent		Referent	
Endorsed	399	23.4	1.13	0.67, 1.89	1.02	0.63, 1.64	0.73	0.33, 1.58
Physical assault								
Not endorsed	1274	74.8	Referent		Referent		Referent	
Endorsed	404	23.7	**2.65**	**1.57, 4.44**	1.39	0.85, 2.26	2.03	0.99, 4.14
Disabling injury/illness								
Not endorsed	1304	76.5	Referent		Referent		Referent	
Endorsed	375	22.0	**2.26**	**1.30, 3.93**	1.40	0.85, 2.27	**2.88**	**1.42, 5.81**
Other life events^d^								
Not endorsed	691	40.6	Referent		Referent		Referent	
Endorsed	1000	58.7	1.08	0.67, 1.73	0.86	0.57, 1.30	0.88	0.42, 1.83
Behavioral characteristics, mental health, and comorbid conditions								
Body mass index, kg/m^2^								
Healthy	617	36.2	Referent		Referent		Referent	
Overweight	771	45.3	1.14	0.71, 1.82	1.32	0.91, 1.90	0.77	0.32, 1.84
Obese	293	17.2	1.24	0.67, 2.28	**1.67**	**1.00, 2.76**	1.12	0.34, 3.62
Smoking status								
Never	683	40.1	Referent		Referent		Referent	
Former smoker	420	24.7	1.00	0.62, 1.61	1.20	0.81, 1.78	1.52	0.77, 2.98
Current smoker	546	32.0	0.87	0.54, 1.38	0.92	0.60, 1.42	1.33	0.51, 3.42
Alcohol problems								
None	1134	66.6	Referent		Referent		Referent	
Positive for alcohol problems	549	32.2	1.51	0.99, 2.29	0.90	0.59, 1.36	1.72	0.96, 3.06
Sleep duration, hours								
< 5	466	27.4	1.24	0.61, 2.48	0.76	0.43, 1.34	1.76	0.60, 5.15
5 to < 7	835	49.0	0.86	0.45, 1.63	1.06	0.64, 1.73	1.25	0.47, 3.26
7 to < 9	171	10.0	Referent		Referent		Referent	
≥9	192	11.3	1.14	0.50, 2.61	0.72	0.35, 1.48	**2.75**	**1.00, 7.48**
Social support^e^								
Not bothered	269	15.8	Referent		Referent		Referent	
Bothered a little	506	29.7	**1.92**	**1.06, 3.44**	0.89	0.54, 1.48	1.63	0.64, 4.09
Bothered a lot	914	53.6	**3.49**	**1.85, 6.54**	0.83	0.42, 1.59	**4.14**	**1.12, 15.14**
Other anxiety syndrome								
Negative	842	49.4	Referent		Referent		Referent	
Positive	812	47.7	**4.27**	**2.42, 7.49**	0.93	0.45, 1.91	**7.40**	**2.25, 24.05**
Bodily pain^b,f^								
Continuous (scaled from 0 to 4)	3.52 (0.24)		**1.86**	**1.44, 2.39**	1.14	0.93, 1.39	1.02	0.72, 1.44
Somatic symptoms								
0–9	469	27.5	Referent		Referent		Referent	
10–14	611	35.9	0.82	0.49, 1.37	1.24	0.81, 1.90	0.85	0.37, 1.95
≥ 15	613	36.0	**1.84**	**1.08, 3.12**	1.09	0.65, 1.80	1.84	0.80, 4.19

*aOR* adjusted odds ratio, *CI* confidence interval; Bolded values are statistically significant with all *p*-values<0.05

^a^ Some variables do not add up to 1704 due to missing data. Full information maximum likelihood was performed to account for missing data and estimate values based on all data that was available

^b^ Values are expressed as mean (standard deviation)

^c^ Deployment dates based on data from Defense Manpower Data Center. Combat based on positive endorsement of any of the following combat exposures: witnessing death due to war, disaster, or tragic event; or witnessing instances of physical abuse, dead and/or decomposing bodies, maimed soldiers or civilians, or prisoners of war or refugees

^d^ Endorsement of any of the following stressful life events: divorce, finances, or sexual harassment

^e^ Measured based on Patient Health Questionnaire question, “In the last 4 weeks, how much have you been bothered by having no one to turn to when you have a problem?”

^f^ Higher score indicates more bodily pain

## Discussion

This current study is among the first, to our knowledge, to longitudinally examine the patterns of PTSD and MDD symptoms across approximately 15 years of follow-up among those with probable comorbid PTSD/MDD at baseline. Four distinct classes of probable comorbid PTSD/MDD were identified among service members and veterans, which illustrate how these comorbid symptoms move in relation to one another over time. The 4 identified classes of PTSD/MDD are consistent with other research that examined trajectories of PTSD and depression separately [23, 48, 49], but uniquely highlight how the symptoms of these comorbid conditions move in tandem with one another.

The largest of the 4 classes (32.3%) included participants who remitted symptoms for both PTSD and MDD and remained at a low level of symptoms throughout the study period. Further, 14.4% of participants gradually remitted symptoms for PTSD and MDD. Participants in these classes had less comorbid health conditions and, on average, were less likely to experience childhood trauma and stressful life events. Alternatively, almost 25% of participants maintained chronically high levels of comorbid PTSD/MDD symptoms throughout the entire study period. This high level of persistence of symptoms is consistent with previous literature, which indicates that symptoms of PTSD and depression often endure for many years, sometimes decades [50]. Yet, prior findings suggest individuals with comorbid PTSD/MDD are more likely to have persistent symptoms than those with only PTSD or MDD [17]. In a previous study, results showed that service members with PTSD who had comorbid MDD were more likely to have persistent PTSD up to 6 years after initially screening positive [17]. This persistence of symptoms highlights the need to identify effective treatments for those with comorbid PTSD/MDD and better understand factors associated with membership in classes of comorbid PTSD/MDD—particularly those with chronic symptoms. Further, previous PTSD trajectory work has highlighted the benefit of tailoring interventions and treatment for PTSD based on the specific trajectory patterns of the individual [51, 52]. Specifically, Galantzer et al. 2013 found that early treatment affected symptom remittance for those who were in the slow recovery class, but not for those in a rapid remittance trajectory [51]. Using a more integrative medicine approach that accounts for the whole person and addresses other mental and physical conditions may help reduce symptoms and lead to less comorbidity over time [53, 54].

Restriction of the sample to individuals meeting criteria for probable PTSD and MDD precluded the formation of many typical classes (e.g., delayed onset, resilience) found in prior studies [18]. Rather than two trajectories (chronicity and recovery) describing individuals with high symptoms, the current study identified four different trajectories of change among individuals with probable comorbid PTSD/MDD. Additionally, even for trajectories that are similar to those found previously (i.e., chronic and gradual recovery trajectories), the level of symptoms reported in the current study was higher than in prior studies [19–21]. This suggests poorer prognoses of individuals with probable comorbid PTSD/MDD. Even within the rapid recovery trajectory, which is most comparable to resilience trajectories, participants continued to report a moderate level of symptoms across the study period. However, similar to prior studies, the proportion exhibiting this optimal trajectory was most prevalent, however, the prevalence (32.3%) was much lower than those exhibiting resilience in prior investigations (80–90%) among those with and without mental health symptoms.

A number of physical, psychosocial, and life experience factors were associated with a chronic course of probable comorbid PTSD/MDD symptoms. In terms of physical factors, those with more bodily pain and/or somatic symptoms were more likely to have chronic symptoms of PTSD/MDD. Previous research indicates that both PTSD and depression affect chronic pain. Comorbid physical symptoms at baseline are strongly associated with new-onset and persistence of PTSD [17, 55, 56]. These physical factors are modifiable through multidisciplinary treatment and care.

The psychosocial factors associated with higher odds of membership in the chronic PTSD/MDD class were anxiety and social support. Those with anxiety were more likely to have chronic symptoms compared with those who recovered rapidly. This highlights that many people suffer from multiple overlapping mental health conditions that may impact treatment and long-term symptoms. Further, those who reported being bothered either “a little” or “a lot” by not having enough social support also had higher odds of membership in the chronic comorbid PTSD/MDD trajectory. This finding is consistent with previous literature on the role of social support in new-onset PTSD and depression [57, 58] as well as persistent symptoms for PTSD [17]. Given this, it is crucial that individuals receive support during their time in service and during and after their transition out of service. Command leaders should also emphasize the need for and importance of social support. Together, these findings further highlight the interwoven nature of physical and mental health and point to the need to screen for co-occurring psychological and physical conditions in order to identify the best ways to address chronicity of comorbid PTSD/MDD symptoms among service members and veterans.

In addition to physical and psychosocial factors, stressful life events/experiences also predicted elevated probable comorbid PTSD/MDD symptoms over time. Specifically, combat experiences, disabling injury/illness, physical assault, and childhood trauma were associated with comorbid PTSD/MDD. Consistent with previous literature, deployment with combat was associated with elevated symptoms over time. Prior evidence clearly shows that combat deployment is associated with new-onset PTSD [59] and depression [60], and with persistent PTSD symptoms among military service members and veterans [17]. Those with a disabling illness/injury and who had experienced physical assault were more likely to have chronic symptoms compared with those who recovered rapidly. Experiencing childhood trauma also significantly increases risk for development of depression and PTSD beyond the role of combat among military service members [36, 61]. Childhood trauma was associated with higher odds of membership in the chronic and relapse trajectories in this study. However, once adjusting for other items, including combat, the association between childhood trauma and comorbid symptoms were attenuated and only remained significant with respect to the relapse trajectory. This indicates that other factors, such as combat and more recent life events, may be more directly associated with elevated symptoms of PTSD/MDD. Although life events are not modifiable, responses to these experiences are adaptable and programs should focus on proper support and treatment for combat- and life-related factors that may impact chronic symptoms.

### Limitations and strengths

Results from this study should be interpreted with several limitations in mind. The Millennium Cohort Study sample may not fully reflect the population of military service members and veterans. However, previous studies have found the cohort to be highly representative of service members and veterans overall [62, 63]. The relatively long 3-year follow-up period between assessments may not fully capture short-term fluctuations in symptoms for PTSD or MDD. However, given the high number of participants who maintained a high level of symptoms over time, and consistency of symptoms throughout the study period, we might not expect to find significant changes on average in PTSD or MDD symptoms over a shorter period of time. We were not able to fully assess differences in trajectories between active duty, Reservist/National Guardsmen, and veterans given changes in status over time. Given this, future studies should explore differences in trajectories between these groups. The self-reported survey data used for analysis may be subject to both recall and reporting bias that could affect the findings. Furthermore, the PTSD Checklist–Civilian Version and PHQ-8 are screening tools for PTSD and MDD, respectively, and are not diagnostic of either condition. Both tools, however, have demonstrated high validity in military and veteran samples, and the items map onto diagnostic criteria [31, 34, 41, 64, 65]. One notable limitation was our inability to assess treatment received for PTSD and/or MDD throughout the study period. The Millennium Cohort Study did not include measures of treatment duration or type for either PTSD or MDD, so it was not possible to examine the effectiveness of treatment received, or how health care utilization might impact long-term comorbidity of PTSD and MDD. However, a study on PTSD symptom trajectories found that treatment received did not affect symptom class membership [51]. Future studies should be conducted to determine how treatment impacts trajectories of comorbid PTSD and MDD.

This study also has many notable strengths, including the large sample of service members and veterans with probable comorbid PTSD and MDD and our ability to examine trajectories of symptoms for both conditions over a duration of approximately 15 years. Furthermore, this study screened both individuals who may and may not seek treatment, which can help identify those at risk for chronic symptoms but might not receive necessary services. The Millennium Cohort Study includes service members and veterans from all service branches and components, which enhances the generalizability of study findings. Further, our broad assessment allows for examination of multiple factors that may be associated with worse prognosis for comorbid PTSD and MDD. Additionally, the current study followed participants during and after service, which is a notable strength, as Department of Defense studies typically only examine during-service outcomes and Department of Veterans Affairs studies usually only examine post-service outcomes.

## Conclusions

Previous research indicates the comorbidity of PTSD/MDD is common and associated with worse sequelae compared to either condition alone. Results from this study indicate that comorbid PTSD and MDD symptoms move in concert with one another and, although many service members and veterans seem to rapidly recover from comorbid PTSD/MDD symptoms, one-quarter of individuals reported high levels of both PTSD and MDD symptoms over time. Due to the high level of persistence and the tandem nature of these 2 conditions, it seems imperative that effective treatment is developed to address comorbid PTSD/MDD. Moreover, those with other mental disorders and physical conditions may have increased risk of chronic symptoms or slow recovery. Among those with comorbid PTSD/MDD, multidisciplinary or integrated medical care that targets both co-occurring mental and physical health conditions may be essential to help reduce symptoms of comorbid PTSD and MDD.

## Supplementary information

**Additional file 1: Figure S1.** Joint trajectories of comorbid posttraumatic stress disorder (PTSD) and major depressive disorder (MDD) adjusted for covariates in the model removing overlapping items from the PCL-C (Loss of interest in things that you used to enjoy; Having difficulty concentrating; Trouble falling or staying asleep) and PHQ-8 (Little interest or pleasure in doing things; Trouble falling or staying asleep, or sleeping too much; Trouble concentrating on things, such as reading the newspaper or watching television). Black indicates trajectories for MDD and Grey indicates trajectories for PTSD. All participants screened positive for comorbid PTSD/MDD at time 1. Each time point is approximately 3 years apart.

## Abbreviations

DSM-IV-TR: Diagnostic and Statistical Manual of Mental Disorders, Fourth Edition, Text Revision
MDD: Major Depressive Disorder
PCL-C: PTSD Checklist–Civilian Version
PHQ: Patient Health Questionnaire
PTSD: Post Traumatic Stress Disorder
VIF: Variance Inflation Factors
