# Supplementary material for: Longitudinal trajectories of comorbid PTSD and depression symptoms among U.S. service members and veterans
Source: BMC Psychiatry. 2019 Dec 13;19:396. doi: 10.1186/s12888-019-2375-1 (PMC6911296; doi:10.1186/s12888-019-2375-1)
Supplement: Supplementary file 1 — Additional file 1: Figure S1. Joint trajectories of comorbid posttraumatic stress disorder (PTSD) and major depressive disorder (MDD) adjusted for covariates in the model removing overlapping items from the PCL-C (Loss of interest in things that you used to enjoy; Having difficulty concentrating; Trouble falling or staying asleep) and PHQ-8 (Little interest or pleasure in doing things; Trouble falling or staying asleep, or sleeping too much; Trouble concentrating on things, such as reading the newspaper or watching television). Black indicates trajectories for MDD and Grey indicates trajectories for PTSD. All participants screened positive for comorbid PTSD/MDD at time 1. Each time point is approximately 3 years apart. [file 12888_2019_2375_MOESM1_ESM.docx]

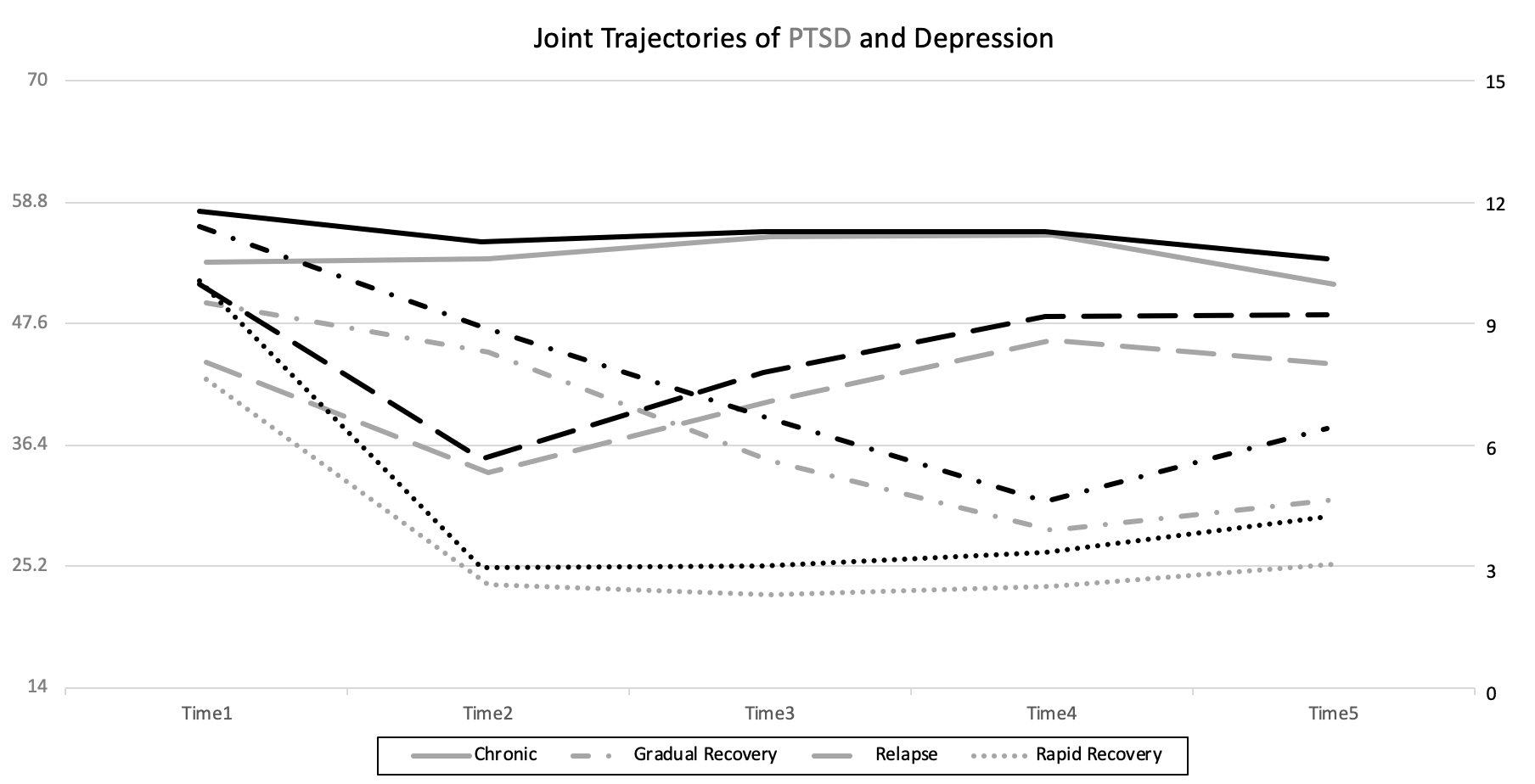
**Supplemental figure 1.** Joint trajectories of comorbid posttraumatic stress disorder (PTSD) and major depressive disorder (MDD) adjusted for covariates in the model removing overlapping items from the PCL-C (Loss of interest in things that you used to enjoy; Having difficulty concentrating; Trouble falling or staying asleep) and PHQ-8 (Little interest or pleasure in doing things; Trouble falling or staying asleep, or sleeping too much; Trouble concentrating on things, such as reading the newspaper or watching television). Black indicates trajectories for MDD and Grey indicates trajectories for PTSD. All participants screened positive for comorbid PTSD/MDD at time 1. Each time point is approximately 3 years apart.

32.3%

29.1%

24.2%

14.4%

PHQ-8 Total Score

PCL-C Total Score

Abbreviations: PCL-C, PTSD Checklist–Civilian Version; PHQ-8: Patient Health Questionnaire 8-item depression scale.

Gray line: PTSD. Black line: MDD.
